# Reemerging Influenza Virus Infections during the Dominance of the Omicron SARS-CoV-2 Variant in Mexico

**DOI:** 10.3390/pathogens11101181

**Published:** 2022-10-13

**Authors:** Mónica Ríos-Silva, Xóchitl Trujillo, Miguel Huerta, Verónica Benites-Godínez, José Guzmán-Esquivel, Jaime Alberto Bricio-Barrios, Oliver Mendoza-Cano, Agustín Lugo-Radillo, Efrén Murillo-Zamora

**Affiliations:** 1 University Center for Biomedical Research, CONACyT—University of Colima, Av. 25 de Julio 965, Col. Villas San Sebastián, Colima 28045, Mexico; 2 University Center for Biomedical Research, University of Colima, Av. 25 de Julio 965, Col. Villas San Sebastián, Colima 28045, Mexico; 3 Health Education Coordination, Mexican Institute of Social Security, Calzada del Ejercito Nacional 14, Col. Fray Junípero Serra, Tepic 63160, Mexico; 4 Academic Unit of Medicine, Autonomous University of Nayarit, Ciudad de la Cultura Amado Nervo, Tepic 63155, Mexico; 5 Faculty of Medicine, University of Colima, Av. Universidad 333, Col. Las Víboras, Colima 28040, Mexico; 6 Clinical Epidemiology Research Unit, Mexican Institute of Social Security Institute, Instituto Mexicano del Seguro Social, Av. Lapislázuli 250, Col. El Haya, Villa de Álvarez 28984, Mexico; 7 Faculty of Civil Engineering, University of Colima, km. 9 carr. Colima-Coquimatlán, Coquimatlán 28400, Mexico; 8 Faculty of Medicine and Surgery, CONACyT—Autonomous University Benito Juarez of Oaxaca, Ex Hacienda Aguilera S/N Sur, camino a San Felupe del Agua, Oaxaca 68020, Mexico; 9 Family Medicine Unit No. 19, Department of Epidemiology, Mexican Institute of Social Security, Av. Javier Mina 301, Col. Centro, Colima 28000, Mexico

**Keywords:** influenza, human, incidence, COVID-19, SARS-CoV-2 variants

## Abstract

The burden of influenza in Mexico has been high. We aimed to characterize its epidemiological patterns before and during the coronavirus disease 2019 (COVID-19) pandemic. A retrospective cohort study was conducted and 5652 PCR-confirmed cases of influenza (October 2019–April 2022) were analyzed. The highest incidence (144 per million) was observed in December 2019 and rapidly decreased right before the start of the pandemic (February 2020). No cases were documented in the 2020–2021 season, and infections reemerged at a low level (8 per million) in December 2021. The case-fatality rates were around 5% in both seasons (*p* = 0.591). The dominant strains were AH1N1 and AH3N2 in the 2019–2020 and 2021–2022 seasons, respectively. In multiple analysis, males and older patients were at increased risk of a fatal outcome. Flu vaccination and infection by B lineages (vs. AH1N1) showed a protective effect. Our results suggest that the spread of the influenza virus reemerged in the 2021–2022 season when the SARS-CoV-2 Omicron variant (B.1.1.529) was dominant. Efforts focusing on the prevention of transmission of respiratory viral pathogens, together with flu vaccination, may be useful to reduce the risk of an influenza outbreak.

## 1. Introduction

In the Northern Hemisphere, the flu season can begin as early as October and can last as late as April or May. The influenza-related burden of disease in Mexico has been historically high [1]. However, the nonpharmaceutical interventions that were taken to prevent the coronavirus disease 2019 (COVID-19) pandemic caused by the severe acute respiratory syndrome coronavirus 2 (SARS-CoV-2) have also decreased the spread of common respiratory viral pathogens, including the influenza virus [2].

The relaxation of interventions combating the COVID-19 pandemic may modify the epidemiological patterns of influenza infections and even lead to a large disease outbreak [3]. In Mexico, the SARS-CoV-2 Omicron variant (B.1.1.529) has been dominant since the end of December 2021 [4], and, even when the virus is able to evade neutralizing antibodies [5], the illness is associated with milder symptoms when compared with previous variants or the wild-type infection.

The objective of this study was to characterize the epidemiology, in terms of incidence rates and case-fatality rates (CFRs), and the dominant viral strain of seasonal influenza in Mexico before and during the COVID-19 pandemic (2019–2022). In addition, we evaluated factors determining the risk of a fatal outcome in patients with laboratory-confirmed influenza virus infection.

## 2. Materials and Methods

We conducted a nationwide retrospective cohort study in Mexico from May to August 2022. Individuals with laboratory-confirmed (reverse-transcription polymerase chain reaction, RT-PCR) influenza infection and symptom onset from October 2019 to April 2022 were enrolled. Eligible patients were identified from the nominal records of a normative system for the epidemiological surveillance of respiratory viral pathogens that belongs to the Mexican Institute of Social Security (IMSS—the Spanish acronym). Clinical files of patients are the main data source for the audited epidemiological surveillance system.

According to normative standards, nasopharyngeal or oropharyngeal swabs were taken from patients with suggestive symptoms of respiratory viral disease. The latest (updated on April 2022) definition of respiratory viral disease includes the presence of at least one major symptom (fever, cough, headache, or dyspnea) together with at least one minor symptom (myalgias, arthralgias, odynophagia, chills, thoracic pain, rhinorrhea, polypnea, anosmia, dysgeusia, or conjunctivitis) [6]. Molecular testing was performed on these biological samples to identify the viral pathogen involved (influenza virus, severe acute respiratory syndrome coronavirus 2 (SARS-CoV-2), or other).

The molecular diagnosis took place in any of four regional (Mexico City, Nuevo León, Jalisco, and Yucatán), highly specialized laboratories of the IMSS. A broader description of the employed laboratory methods has been published elsewhere [7].

Enrolled subjects were classified, according to the date of symptom onset, into one of the following influenza seasons (October–April): 2019–2020, 2020–2021, and 2021–2022. Summary statistics were computed. Seasonal and age-stratified (0–9, 10–19, 20–49, 50–59, and 60-or-above years old) incidence rates (per million people) and case-fatality rates (CFRs, per 1000) were obtained. We used risk ratios (RRs) and 95% confidence intervals (CIs) to evaluate factors associated with death risk, and they were computed through a multiple generalized linear regression model. 

## 3. Results

Data from 5652 laboratory-confirmed cases of influenza were analyzed. No laboratory-confirmed cases were registered in the 2020–2021 season. The proportions of enrolled cases in the remaining seasons were as follows: 2019–2020, 85.6% (*n* = 4836); 2021–2022, 14.4% (*n* = 816).

Most of the analyzed patients were female (54.7%) and the mean age (± standard deviation) was 32.5 ± 20.8 years old (total range: 0–95 years). No significant differences in the mean ages of patients were observed between the analyzed influenza seasons (*p* = 0.180). Table 1 shows the characteristics of the study sample for selected variables.

**Figure 1:**
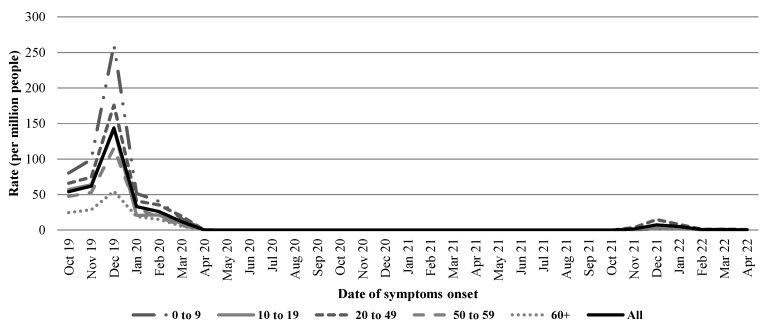
Incidence rates (per million people) of laboratory-confirmed cases of influenza in Mexico, 2019–2022.

As can be observed in Figure 1, the highest incidence rate (260 cases per million) was observed in December 2019 among children aged 0–9 years old. The overall rate during the same period was 144 cases per million and rapidly decreased during January 2020. Influenza transmission continued at low levels during the rest of the season (February–April 2020).

In the latest analyzed season (2021–2022), the highest rate (8 cases per million) was also registered in the month of December, and young adults (20–49 years old) had the highest age-stratified rate (15 cases per million).

A total of 245 fatal outcomes were registered in 2019–2020, this figure being almost seven times higher than that documented for the influenza 2021–2022 season (*n* = 45). The cumulative CFRs were 5.1% and 5.5% in the first and last influenza seasons, respectively (*p* = 0.591).

**Figure 2:**
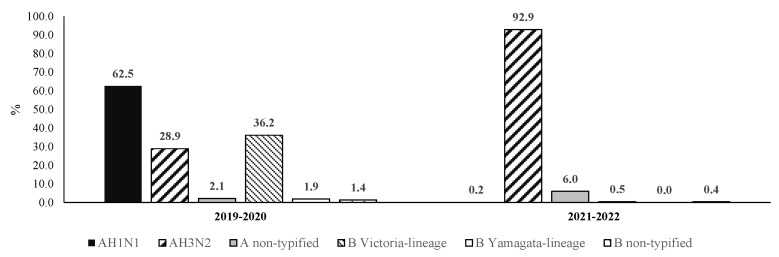
Proportion of viral strains identified in laboratory-confirmed cases of influenza (Mexico, 2019–2022).

The AH1N1 strain was predominant in the 2019–2020 season, and it was isolated in nearly two-thirds (62.5%) of cases (Figure 2). The B Victoria lineage and AH3N2 strains were identified in 36.2% and 28.9% of patients, respectively. During the latest influenza season (2021–2022), AH3N2 became clearly predominant and caused more than nearly 93% of infections. In addition, during the 2021–2022 season, the AH1N1 and B Victoria lineage strains were isolated in 2 and 5 out of 1000 PCR-confirmed cases, respectively.

In multiple regression analysis (Table 2), the 2021–2022 influenza season (vs. 2019–2020) was not associated with significant increased risk of dying. Factors associated with a higher risk of a fatal disease outcome were male gender (RR = 1.02, 95% CI 1.01–1.03) and older age, particularly being aged 40–59 years old (vs. 0–9: RR = 1.07, 95% CI 1.05–1.09) or 60 years or above (RR = 1.23, 95% CI 1.20–1.27). As also shown in Table 2, flu vaccination (RR = 0.97, 95% CI 0.96–0.99) and B lineages (vs. AH1N1: Victoria, RR = 0.96, 95% CI 0.95–0.98; Yamagata, RR = 0.95, 95% CI 0.91–0.99) were associated with decreased risk of a fatal outcome.

## 4. Discussion

Our study characterized epidemiological aspects related to influenza virus infections in Mexico right before and during the COVID-19 pandemic. The presented results suggest that the disease patterns have been heterogeneous during the pandemic waves in terms of incidence, fatality risk, and predominant viral strains.

We observed a decrease in the incidence of PCR-confirmed influenza infections coincident with the beginning of the COVID-19 pandemic in Mexico (Figure 1). This decreasing trend was previously documented in our country and other territories [8,9,10]. The first COVID-19 case in Mexico had been registered by the end of February 2020 [11]. Factors determining this scenario may have included increased fear of getting sick and dying from COVID-19, which may in turn have increased adherence to nonpharmaceutical preventive interventions, such as hand hygiene and use of face masks. Low adherence to these practices was described during influenza seasons before the coronavirus pandemic [12].

Interestingly, no influenza cases were documented during the 2020–2021 season or when the Delta variant was dominant [6]. We hypothesize that this may have been determined, at least partially, by the re-implementation of strict physical distancing and other nonpharmaceutical interventions during the Delta variant’s emergence and an increase in the proportion of the population requesting seasonal influenza vaccination [13,14].

In our analysis, influenza cases had reemerged by December 2021 simultaneously with the replacement of Delta with Omicron as the dominant variant. Since this latter variant was associated with milder symptoms [15], a relaxation of strategies reducing the spread of respiratory viral pathogens may have been involved in the re-emergence of the influenza virus. These relaxed strategies may have included the lockdown, travel restrictions, and personal protective measures (such as the widespread use of masks) [16,17], which targeted the prevention and control of SARS-CoV-2 transmission. It is also important to cite that the observed incidence rates in the 2021–2022 season were considerably lower than those registered in 2019–2020.

Another major finding from our study is that the predominant viral strains changed and that in the 2021-2022 season AH3N2 replaced AH1N1 (2019–2020) as the dominant strain. The same scenario has been observed in the rest of the Northern Hemisphere [18] and is likely to be related to the biannual behavior of viral subtypes [19]. Despite the strain variations, the CFRs were similar (*p* = 0.591) in both influenza seasons. In the Southern Hemisphere, where flu seasons usually occur from April to September, a reemergence of influenza virus infections was also observed with the predominance of the AH3N2 subtype [20].

The limitations of this study must be cited. Since we used secondary data from an epidemiological surveillance system, we were unable to ensure that there were no influenza cases in the 2020–2021 season. It seems more plausible that cases occurred but at a low level such that they were not identified, for some reason, as influenza cases. Influenza activity in the rest of the Northern Hemisphere in the 2020–2021 period was reported as low [21].

## 5. Conclusions

Our results suggest that the transmission of the influenza virus reemerged during the 2021–2022 season, coincident with the dominance of the Omicron variant of SARS-CoV-2 and the relaxation of non-pharmaceutical interventions implemented earlier in the COVID-19 pandemic. Therefore, an influenza outbreak might be feasible and preventive interventions may be highly useful to reduce the risk.

## Figures and Tables

**Table 1:** Characteristics of the study sample for selected variables (Mexico, 2019–2022).

Characteristic	Overall, *n* (%)		Flu Season, *n* (%)				*p*
			2019–2020		2021–2022		
Gender							
Female	3099	(54.8)	2633	(54.5)	466	(57.1)	0.158
Male	2553	(45.2)	2203	(45.5)	350	(42.9)	
Age group (years)							
0 to 9	997	(17.6)	955	19.8	42	5.2	<0.001
10 to 19	504	(8.9)	452	9.4	52	6.4	
20 to 39	2371	(42.0)	1927	39.9	444	54.4	
40 to 59	1122	(19.9)	972	20.1	150	18.4	
60 or above	658	(11.6)	530	11.0	128	15.7	
Flu-vaccinated ^a^							
No	4710	(83.3)	3979	82.3	731	89.6	<0.001
Yes	942	(16.7)	857	17.7	85	10.4	
Pneumonia ^b^							
No	5051	(89.4)	4292	88.75	759	93.01	<0.001
Yes	601	(10.6)	544	11.25	57	6.99	
Hospital admission							
No	3197	(56.6)	2610	54.0	587	71.9	<0.001
Yes	2455	(43.4)	2226	46.0	229	28.1	
Disease outcome							
Recovery	5362	(94.9)	4591	94.9	771	94.5	0.591
Death	290	(5.1)	245	5.1	45	5.5	

Note: No cases were documented during the 2020–2021 influenza season. ^a^ During the same season of symptoms onset. ^b^ Pneumonia cases were defined by clinical and radiographic findings suggestive of this alteration in inpatients with laboratory-confirmed influenza infection.

**Table 2:** Characteristics associated with mortality risk due to laboratory-confirmed seasonal influenza (Mexico, 2019–2022).

Characteristic	RR (95% CI), *p*			
	Bivariate Analysis		Multiple Analysis	
Gender				
Female	1.00		1.00	
Male	1.02 (1.01–1.03)	0.004	1.02 (1.01–1.03)	0.004
Age group (years)				
0 to 9	1.00		1.00	
10 to 19	0.99 (0.98–1.02)	0.974	1.01 (0.98–1.03)	0.765
20 to 39	0.99 (0.98–1.01)	0.846	1.01 (0.99–1.02)	0.693
40 to 59	1.08 (1.06–1.10)	<0.001	1.07 (1.05–1.09)	<0.001
60 or above	1.23 (1.21–1.26)	<0.001	1.23 (1.20–1.27)	<0.001
Influenza season of symptom onset ^a^				
2019–2020	1.00		1.00	
2021–2022	1.01 (0.99–1.02)	0.588	1.01 (0.99–1.03)	0.211
Flu-vaccinated ^b^				
No	1.00		1.00	
Yes	0.96 (0.95–0.98)	<0.001	0.97 (0.96–0.99)	<0.001
Identified virus subtype				
AH1N1	1.00		1.00	
AH3N2	0.96 (0.94–0.97)	<0.001	0.96 (0.95–0.98)	<0.001
A non-typified	0.98 (0.93–1.02)	0.272	0.98 (0.94–1.01)	0.161
B Victoria lineage	0.93 (0.92–0.94)	<0.001	0.96 (0.95–0.98)	<0.001
B Yamagata lineage	0.96 (0.91–1.01)	0.128	0.95 (0.91–0.99)	0.046
B non-typified	0.97 (0.92–1.02)	0.262	0.97 (0.92–1.03)	0.335

Notes: (1) Generalized linear regression models were used to estimate risk ratios (RRs) and 95% confidence intervals (CIs); (2) the estimates from the multiple model were adjusted by all the variables listed in the table. ^a^ According to the Northern Hemisphere (from October to April of the next year). Since no laboratory-confirmed cases of influenza were documented in the 2020–2021 season, it was excluded from the regression analysis. ^b^ During the same season of symptom onset.

## Data Availability

The data presented in this study are available on request from the corresponding author.
